# Experimental Evaluation of a UWB-Based Cooperative Positioning System for Pedestrians in GNSS-Denied Environment

**DOI:** 10.3390/s19235274

**Published:** 2019-11-29

**Authors:** Jelena Gabela, Guenther Retscher, Salil Goel, Harris Perakis, Andrea Masiero, Charles Toth, Vassilis Gikas, Allison Kealy, Zoltán Koppányi, Wioleta Błaszczak-Bąk, Yan Li, Dorota Grejner-Brzezinska

**Affiliations:** 1 Department of Electrical and Electronic Engineering, The University of Melbourne, Victoria 3010, Australia; liy19@student.unimelb.edu.au; 2 Department of Geodesy and Geoinformation, TU Wien-Vienna University of Technology, 1040 Vienna, Austria; 3 Department of Civil Engineering, Indian Institute of Technology, Kanpur 208016, India; sgoel@iitk.ac.in; 4 School of Rural and Surveying Engineering, National Technical University of Athens, 157 73 Athens, Greece; hperakis@central.ntua.gr (H.P.); vgikas@central.ntua.gr (V.G.); 5 Interdepartmental Research Center of Geomatics, University of Padova, 35020 Legnaro, Italy; masiero@dei.unipd.it; 6 Department of Civil, Environmental and Geodetic Engineering, The Ohio State University, Columbus, OH 43210, USA; toth.2@osu.edu; 7 Department of Geospatial Science, RMIT University, Melbourne, Victoria 3000, Australia; allison.kealy@rmit.edu.au; 8 Leica Geosystems, 9435 Heerbrugg, Switzerland; zoltan.koppanyi@gmail.com; 9 Institute of Geodesy of the University of Warmia and Mazury, 11-041 Olsztyn, Poland; wioleta.blaszczak@uwm.edu.pl; 10 College of Engineering, The Ohio State University, Columbus, OH 43210, USA; grejner-brzezinska.1@osu.edu

**Keywords:** cooperative positioning, extended kalman filter, Indoor positioning, relative ranging, ultra-wideband

## Abstract

Cooperative positioning (CP) utilises information sharing among multiple nodes to enable positioning in Global Navigation Satellite System (GNSS)-denied environments. This paper reports the performance of a CP system for pedestrians using Ultra-Wide Band (UWB) technology in GNSS-denied environments. This data set was collected as part of a benchmarking measurement campaign carried out at the Ohio State University in October 2017. Pedestrians were equipped with a variety of sensors, including two different UWB systems, on a specially designed helmet serving as a mobile multi-sensor platform for CP. Different users were walking in stop-and-go mode along trajectories with predefined checkpoints and under various challenging environments. In the developed CP network, both Peer-to-Infrastructure (P2I) and Peer-to-Peer (P2P) measurements are used for positioning of the pedestrians. It is realised that the proposed system can achieve decimetre-level accuracies (on average, around 20 cm) in the complete absence of GNSS signals, provided that the measurements from infrastructure nodes are available and the network geometry is good. In the absence of these good conditions, the results show that the average accuracy degrades to meter level. Further, it is experimentally demonstrated that inclusion of P2P cooperative range observations further enhances the positioning accuracy and, in extreme cases when only one infrastructure measurement is available, P2P CP may reduce positioning errors by up to 95%. The complete test setup, the methodology for development, and data collection are discussed in this paper. In the next version of this system, additional observations such as the Wi-Fi, camera, and other signals of opportunity will be included.

## 1. Introduction

Positioning in Global Navigation Satellite Systems (GNSS)-denied/challenged indoor/outdoor and transitional environments remains one of the most challenging problems due to the presence of various objects that generally reflect and disperse signals used for positioning, and thus affect the network geometry and data availability. Therefore, such environments and situations require new robust approaches for positioning that mitigate some of these challenges. For that purpose, in this paper we deploy an indoor cooperative positioning (CP) system and test its performance.

CP has demonstrated to be useful for positioning of mobile platforms navigating in challenging GNSS, as well as GNSS-denied environments ([1,2,3,4]), where at least some of the nodes can achieve an acceptable level of positioning accuracy using GNSS, while other nodes are in GNSS-denied environment(s), and the nodes in the network are well-connected to each other and inter-node range information is available ([1,2,3,5]). The CP approach relies on information exchange in an inter-connected network of multiple nodes that could be static (called anchor/infrastructure nodes) or dynamic (such as Unmanned Aerial Vehicles (UAVs), pedestrians, cars) in nature ([1,2,3,4,6,7,8,9,10,11,12,13]). Each node shares information about its own state, as well as relative information with respect to its neighbouring nodes. The absolute and relative information received from nodes can be processed to precisely estimate the state (including localisation) of each node. Various CP systems-based on sensors such as Ultra-Wide Band (UWB) [7], Wireless Fidelity (Wi-Fi) ([7,8]), Bluetooth, or other similar sensors ([9,10,11,12,13]) have been developed in the literature. These CP systems can be classified according to the type of sensor observations (such as angle of arrival, or return time trip), type of processing architecture used (centralised vs. distributed) and the presence or absence of static anchors (infrastructure-based vs. anchor-free). Compared to distributed architectures, centralised ones offer improved accuracy but at the cost of increased communication and processing requirements. In contrast, distributed architecture offers robustness, scalability, and better reliability of the cooperative network ([2,9]). In addition, distributed architectures are adaptive—that is, they can take care of addition or loss of nodes from a network. However, one of the major limitations of distributed algorithms is the presence of unknown correlation among the states of the nodes [14]. Various distributed algorithms, such as Belief Propagation (BP) ([9,10,11]) and the Covariance Intersection Filter ([14,15]) have been proposed for CP. Inclusion of a static anchor (i.e., infrastructure) nodes has been shown to improve localisation accuracy [2]. On the other hand, anchor-free (or P2P) cooperative systems do not rely on the presence of a fixed infrastructure and can use ad hoc networks for positioning.

In completely GNSS-denied environments, such as an indoor environment, a CP network can be best utilised by realisation of the sufficient number of static anchor nodes, whose precise location is known in advance. Various authors have demonstrated the use of alternative positioning technologies for positioning in GNSS-denied environments (see, e.g., [6,7,8]). In this paper, a CP approach that is reliant on the presence of a large number of static infrastructure/anchor nodes is referred to as peer-to-infrastructure CP (P2I CP), while a peer-to-peer CP (P2P CP) relies only on the communication and information exchange among dynamic nodes. From a practical standpoint, a P2I CP may offer better accuracy as compared to a P2P CP in challenging environments [16]. However, this is achieved at the cost of increased investment in terms of time and cost required in setting up a large number of static anchor nodes in a P2I framework [2]. Therefore, a good balance with the two extremes (P2P+P2I) may offer reasonable positioning accuracy, especially in indoor and challenging environments, where GNSS signals may be completely absent.

Recent studies ([4,5,17]) on indoor positioning using UWB demonstrate the usefulness and capabilities of both CP approaches, as well as UWB sensors. The achievable accuracy in indoor environments is reported to be from 30 cm to 2 m. The researchers rely either only on anchor-based positioning [4] (i.e., P2I) or use a small number of nodes (in some cases, only up to 2) [17]. In this paper, we aim to show the benefits and capabilities of the centralised P2P+P2I CP under real-world conditions where bad geometry and data availability affect performance. This will be tested on an ad hoc network of 30 infrastructure nodes and for multiple dynamic nodes (i.e., pedestrians). We investigate the use of UWB technologies as an alternative technique for pedestrian CP in an indoor environment. The real-world data used in this paper were collected as part of a benchmarking measurement campaign. The campaign was carried out in the course of the joint International Association of Geodesy (IAG) and the Federation of Surveyors (FIG) working groups’ effort on “Multi-Sensor Systems” at the Ohio State University (OSU) in October 2017 to investigate new approaches and algorithms (more in [1]). In the experiments (described later), pedestrians jointly navigated together to achieve CP ubiquitous positioning. For the pedestrians, a prototype helmet equipped with a variety of sensors, including two UWB systems, was designed. The results demonstrate that P2P+P2I CP techniques can be useful for the positioning of platforms navigating in swarms or networks and offer significant improvement in terms of positioning accuracy. However, some drawbacks have been observed under good geometry and data availability conditions, where P2I CP performs better. Using the two UWB systems, decimetre-level positioning accuracy is achieved under typical real-world conditions.

The major contribution of this paper is that it experimentally evaluates and compares the performance of P2P and P2P+P2I CP approaches in challenging and constrained indoor environments (such as the hallway), where poor network geometry and poor connectivity among the nodes is frequently observed. Furthermore, this paper also presents a new prototype (currently under development) of a Multi-Sensor Ubiquitous Positioning System (MUPS) specially designed for pedestrians in indoor, outdoor, and transitional environments. This paper not only offers a realistic and practical overview of the performance of UWB-based indoor CP systems, but also demonstrates the existing capabilities of proposed MUPS in real-world environments and highlights that some of these challenges may be mitigated once the proposed MUPS is fully developed and functional.

The remainder of the paper is organised as follows: Firstly, the state-of-the-art UWB sensors and positioning are described in Section 2, followed by a description of the experimental schemes and test scenarios in combined indoor/outdoor environments, as well as the sensor specifications and characteristics in Section 3. Section 4 then covers the positioning framework, and Section 5 the description and discussion of the main results and findings. Finally, Section 6 provides a summary and an outlook on future plans for the data-processing and analyses.

## 2. State-of-the-Art in UWB Positioning

The Federal Communications Commission (FCC) defines UWB as a Radio Frequency (RF) signal that has a bandwidth greater than 500 MHz and is generally operated in the 3.1–10.6 GHz range. This high bandwidth of UWBs, allowing the use of short-pulse waveforms, is useful in reducing the effect of interference due to a multipath [18]. The lower part of the frequencies in UWB enables it to penetrate obstacles, including walls and objects. UWB is becoming one of the most sought-after solutions for positioning in GNSS-denied environments because UWB systems offer better multipath resolution capabilities, higher data rates, and lower interference with existing systems, as well as generally having lower energy requirements. The authors in [19] divided the UWB positioning methods into two broad categories—namely, fingerprinting-based and geometric methods. The geometric methods estimate the position either using the range or angle information derived from Received Signal Strength Indicator (RSSI), Time of Arrival (TOA), Time Difference of Arrival (TDOA), or Angle of Arrival (AOA) measurements observed by a UWB system. In contrast, fingerprinting methods rely on a location-dependent "fingerprint" that is matched with an offline fingerprinting database to derive the location of the target. In general, fingerprinting methods use UWB-derived RSSI observations as the input. Fingerprinting methods are generally labour-intensive, as a significant amount of effort is required to develop a comprehensive fingerprint database. This database is then used in normal operations for positioning, where the collected signal is compared against the database to derive the position estimate. Furthermore, in comparison to fingerprinting methods, geometric methods are faster and provide higher localisation accuracy. The remaining section focuses on geometric methods for UWB positioning and provides a review and comparative analysis of these methods.

### 2.1. TOA, RTT, and TDOA-Based Methods

The TOA methods rely on the precise estimation of the time of arrival of signals, which is converted to a range observation between the transmitter and receiver. The stand-alone receiver position (in 3D) can be estimated once range observations from at least three transmitters are known and the positions of all transmitters are known in advance. A multi-lateration approach forms the basis of the position estimation using range measurements. The major limitation of the TOA methods is the requirement of precise synchronisation between the transmitter and receiver. Even relatively small time synchronisation errors between the two can result in significant errors in the derived range measurements, which can have a large impact on the estimated position. To overcome the limitations due to synchronisation, modern UWB systems, including Time Domain and Decawave, make use of a Round Trip Time (RTT) approach to derive the range estimate. These systems measure the total time taken by the signal to travel from the transmitter to the receiver and back to the transmitter. These time measurements also include the time required by the receiver to detect and send the signal back to the transmitter. This time is usually constant and can be obtained from calibration. By doing so, they can bypass the requirement of transmitter and receiver to be in synchronisation with each other. Various estimation algorithms, such as the Extended Kalman Filter (EKF) [2], Particle Filter (PF), least squares with multi-dimensional scaling (LS-MDS) [20], and Generalized Gaussian Mixtures (GGM) [21] have been demonstrated to estimate the receiver position. TOA- and RTT-based methods have been used in both line-of-sight (LOS) and non-line-of-sight (NLOS) environments for various applications, including personnel localisation in coal mines [22], locating body-worn sensors [23], UAV tracking [24], Intelligent Transportation Systems (ITS) [16], and cooperative positioning ([24,25]).

The TDOA-based methods estimate the receiver position by measuring the time difference of arrival between the receiver and multiple transmitters. Consider one receiver and three transmitters *A*, *B*, and *C*. The receiver estimates the TDOA between *A* and *B*, *A* and *C*, and *B* and *C* to derive its own position with respect to the transmitters where to corresponding lines-of-positions are hyperbolas between the transmitters. Unlike TOA methods, TDOA does not require the transmitter and receiver to be synchronised [26]. However, the bandwidth requirement in TDOA-based methods can be significantly higher as compared to other methods [18]. These methods have been demonstrated in health-care applications [27], parking systems [28], tracking in complex indoor environments [29], underground mines [30], indoor navigation [31], robotic applications [32], and so forth. Some of the commercial UWB systems that use the TDOA approach include Ubisense and Decawave.

### 2.2. AOA-Based Methods

The AOA-based methods estimate the angle of signal reception by the receiver from the transmitter, followed by receiver position estimation on the basis of the intersection of the angle line from each of the transmitters [19]. Such methods require installation of an antenna array on each of the nodes and rely on a beamforming approach to derive the angle. The angle of arrival is estimated by measuring the phase difference between elements of the antenna array or power spectral density across the array. The complexity due to the geometry of the antenna array is one of the limiting factors in the adoption of AOA-based methods. Additionally, AOA methods have higher power requirements, can be complex, and are generally expensive. The accuracy of these methods is highly dependent on the distance between the transmitter and receiver and decreases with increasing distances [18]. Higher accuracies can be achieved using AOA methods by increasing the Signal-to-Noise Ratio (SNR), effective bandwidth, size of antenna array, and number of antenna elements [19].

One of the advantages of AOA-based methods is that they require a smaller number of anchor nodes (or transmitters with known position) as compared to TOA, RTT, or TDOA-based methods. Furthermore, AOA methods do not require clock synchronisation between the nodes, unlike TOA/TDOA methods. AOA-based methods have been demonstrated in areas such as cooperative localisation [33] and resource management of construction projects [34]. In addition to their relatively higher costs, the utility of AOA-based methods is severely limited by their complex antenna, especially in low-cost and UAV applications where sensor form-factor and weight are the key important factors. An example of the commercial system that relies on the AOA approach has been developed by Ubisense [35].

### 2.3. RSSI-Based Methods

Geometric-RSSI methods measure the strength of the signal received by the receiver and utilise empirical models to convert the observed signal strength into a range observation between the transmitter and receiver. The derived range observation from three or more transmitters is used to estimate the receiver position using a multi-lateration approach. In ideal environments, RSSI and distance (between receiver and transmitter) are inversely related and can be expressed by a signal power decay model. From a practical standpoint, such simple models are unusable due to noise, multipath, and shadowing [36]. Therefore, a specific class of empirical models called “path-loss models” are commonly used to derive the range estimates using RSS observations. One of the most commonly used models is a log-distance path loss model that expresses the decrease in RSS with distance from the transmitter, and is given by the following equation [37]:

(1) $$PL\left(d\right)=PL\left(d_{o}\right)+10\gamma \log\left(\frac{d}{d_{o}}\right)+S,\;d\geq d_{o}\geq d_{f}.$$

In the above equation, the parameter $S\left(∼N\left[0,\sigma \right]\right)$ is introduced to account for the effects of shadow fading for both the LOS and NLOS paths. The parameters γ, $d_{f}$, and $d_{o}$ denote the pass loss exponent, Fraunhofer distance, and reference distance, respectively. The path losses at distances *d* and $d_{o}$ are denoted by $PL\left(d\right)$ and $PL\left(d_{o}\right)$, respectively, and are expressed in decibels. These methods have been explored for positioning in an indoor environment in both LOS and NLOS conditions ([38,39,40,41]).

It has been demonstrated that the performance of RSS methods in terms of achievable positional accuracy is generally low as compared to methods such as TOA and TDOA [41]. The other factors that restrict the use of RSS-based methods are the complexity of modelling path-loss and suitability of these methods to short-range applications. However, compared to the other methods, RSS-based methods offer certain advantages which make them attractive in special scenarios. These advantages include the relatively low cost of the required hardware, no limitation on the number of nodes that can be used, and less communication traffic in the network.

### 2.4. Hybrid Methods

Hybrid methods make use of two or more types of UWB-derived observations listed in Section 2.1, Section 2.2 and Section 2.3. Although hybrid methods can be complex and expensive compared to the other methods, they often result in higher accuracy compared to these methods when used alone. Hybrid approaches can make use of complementary properties of individual methods and combine the strengths of individual approaches to achieve higher performance. The authors in [41] used a combination of AOA and TDOA observations in conjunction with Inertial Measurement Unit (IMU) measurements to achieve higher accuracy in pedestrian positioning. By using both AOA and TDOA observations, the authors were able to detect the presence of multipath in the range observations. The authors in [42] used a combination of TDOA and RSS observation to develop an indoor/outdoor system for disaster aid. The UWB system was only used indoors, and outdoors positioning was done using GNSS. A combination of AOA and RSS observations was used by the authors in [43] to design a positioning system capable of mitigating NLOS errors. The authors in [44] demonstrated the effectiveness of combining TOA and AOA observations in terms of superior performance and power consumption requirements. Various other hybrid systems have been developed and demonstrated by many authors. For a comprehensive review of these systems, the reader is referred to the publications by [18,19]. The next section presents and explains the experimental setup of cooperative positioning and discusses its characteristics.

## 3. CP Prototype System and Experiences

This section introduces the developed prototype of the multi-sensor ubiquitous positioning system (MUPS) used for performance evaluation of the CP system. This is followed by the description of the test site, experimental conditions, and procedures for data collection. Furthermore, the availability of data under different conditions during the field tests, which significantly affects the positioning performance of the system, is discussed in Section 3.4.

### 3.1. MUPS Prototype

**Figure 1:**
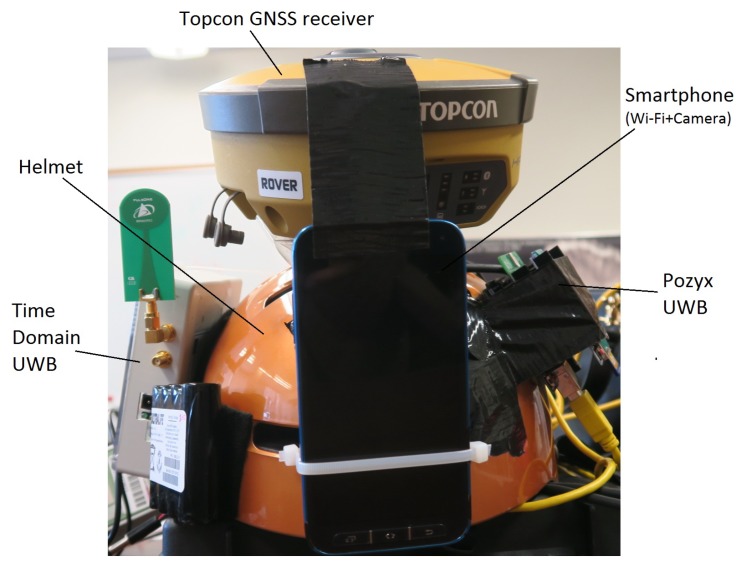
One of the MUPS prototypes with sensors, including a compact GNSS receiver, UWB radios, and a smartphone.

The MUPS prototype, currently under development, is dedicated to achieving ubiquitous positioning in indoor, outdoor, and transitional environments. This prototype integrates a large number of complimentary sensors and signals, including the GNSS receiver, Inertial sensors, UWB radios, Wi-Fi signals, and camera observations. All the sensors are integrated on a helmet, which can be worn by the user. One of the prototypes developed in this research is shown in Figure 1. The GNSS antenna and receiver are installed at the top of the helmet, and two different UWB units are installed on the sides of the helmet. The Wi-Fi and camera observations are recorded by the smartphone which is located towards the front of the user. The sensors are integrated via an on-board processor, that ensures the synchronisation and coordination among multiple sensors. In this research, four such helmet units are developed, all of which are identical. The four helmets can measure relative ranges with respect to each other using either of the two UWB units installed on the helmets, and thus form a cooperative network where the information is shared amongst all the network nodes (i.e., four user helmets). The advantage of this cooperative network is that each node assists the neighbouring nodes in localisation, and thus may improve the positioning accuracy. A more detailed description of the prototype and used sensors can be found in [1].

In addition to including four helmets that act as dynamic nodes, this research uses static anchor nodes as well (about 30 spread through the test site) to form a cooperative network. The static anchor nodes are equipped with either Time Domain or Pozyx UWB radios and are capable of communicating and sharing information with the helmets (i.e., users) moving indoors and outdoors. One of the biggest advantages of including static anchor nodes (i.e., infrastructure nodes) in a cooperative network is that they can improve the localisation accuracy of all the moving nodes in the network, especially in challenging environments [2] and offer a constraint to the ever-changing geometry of mobile nodes [16]. The complete details of the experimental setup, including environmental conditions and locations of static anchor nodes are given in Section 3.2. Although the helmets can record Wi-Fi and camera observations, they are not used in this paper. This paper relies only the observations recorded by UWB sensors. The other sensor observations, such as Wi-Fi and camera, will be integrated into future research.

#### UWB Systems Description

**Figure 2:**
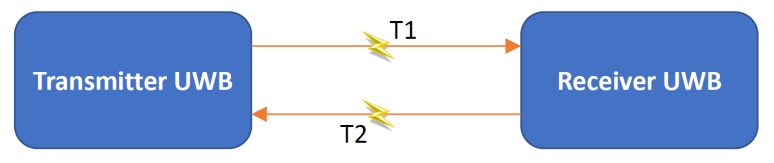
TW-TOF measurement between the transmitter and receiver UWB nodes.

This research used two different UWB systems: Time Domain P410 [45] and P440 [46] and Pozyx [47] in the developed prototype. Both systems measure the two-way time of flight (TW-ToF) (or RTT) from the transmitter to the receiver ($T_{1}$) and back to the transmitter ($T_{2}$) including the time taken by the receiver to respond to the transmitter UWB ($T_{d}$) to derive the range observations between a transmitter and receiver (as shown in Figure 2). The time $T_{d}$ is generally constant and can be estimated using calibration. The range observation, in this case, is given by (2), where *c* denotes the speed of light and $T_{d,calib}$ denotes the estimated value of $T_{d}$ obtained after calibration. A summary of the specifications of both the UWB systems is given in Table 1.

(2) $$r_{observed}=\frac{c}{2}\times \left[\left(T_{1}+T_{2}+T_{d}\right)-T_{d,calib}\right]$$

UWB sensors rely on the coherent (i.e., phase-stable continuous oscillation) transmission of very short-duration Radio Frequency (RF) waveforms (i.e., pulses). UWB transmissions consist of packets of several thousands of pulses, offering the ability to perform high-accuracy range measurements both in LOS and NLOS conditions, even at high multipath operating conditions. Their operating principle is based on TW-ToF/RTT (explained above), in which the total travel time of the RF signal with the process time of the UWB transceivers subtracted is divided by two and multiplied by the RF signal velocity in order to resolve the Precise Range Measurement (PRM) value. In effect, based on RF signals that spread over a large bandwidth, a number of short duration pulses transmitted among the UWB nodes are used for estimating the required travel time of the RF signal. Furthermore, by exploiting the signal characteristics of short pulses, detection of the first pulse (i.e., first break (FB) or leading edge (LE)) is possible with high accuracy, thus enabling the range measurement of the direct signal, while the effects of high multipath signals are simultaneously filtered out.

As indicated above, two Time Domain modules (P410 and P440) have been used. The P410 and P440 Time Domain modules offer high ranging performance with a nominal precision reported better than 2 cm. The nominal accuracy, as reported by the manufacturer [45,46] and validated in prior performance evaluation campaigns [48], is of the order of ∼3 cm for calibrated UWB pairs. It is worth noting that prior to the experiments, the compatibility among the different module versions of Time Domain UWBs was examined exhaustively confirming full interoperability and functionality. During data collection, each UWB module was preprogrammed in order to make either a ranging request, respond to a ranging request, or both. In the setup used in our experiments, the master rover node (i.e., the node that initiated the ranging requests) was connected to a logging Personal Computer (PC) running a custom-built Matlab script performing ranging requests to all infrastructure nodes in the vicinity at a ping frequency of about 2.5 Hz (maximum achieved frequency given the number of Time Domain devices in the network). Time synchronisation is based on the PC time reported at the instant of the ranging response. The internal (on-board) timestamp of the master module was logged as well. The operating distance for all units was examined prior to the trials and determined to be at least 170 m outdoors. This range capability proved sufficient considering that the maximum distance in LOS in the indoor corridor is less than 60 m.

The accuracy of Pozyx range measurements is at a decimetre level. Typical 2D positioning accuracy in good working conditions (i.e., LOS signals, good geometry) is 10–15 cm for static UWBs [49] and 30–50 cm for dynamic nodes [50]. Prior to the data collection experiments, it was determined that interference between Pozyx and Time Domain device signals does not occur given the settings used during the data collection. Furthermore, each dynamic node was preprogrammed to periodically request range measurements from each anchor node in a loop. In the case of the Pozyx devices, the ranging requests to all infrastructure nodes were made at a ping frequency of about 4.3 Hz (maximum achieved frequency given the number of Pozyx devices in the network). The Pozyx devices logged the internal timestamp and were not synchronised to the PC time. The operating distance for Pozyx devices was also tested outdoors in good LOS conditions and it was determined to be around 100 m. Unlike Time Domain UWBs, Pozyx devices are equipped with an IMU (triaxial accelerometer, gyroscope, and magnetometer) and pressure sensor (these sensors were not used in the tests).

Due to the use of two different UWB systems with different internal timestamps, time synchronisation was necessary. One dynamic node was equipped with the Pozyx UWB, and the other one with the Time Domain UWB (synchronised with PC time). The time difference for two devices was determined based on known positions of the dynamic nodes at the same time. All Pozyx UWB’s timestamps were reduced to the Time Domain PC timestamp which is done in offline mode. Since the initial time offset is known, and the data capturing rate of each of the UWB sensors is specified, the time at which data is captured by each UWB can be estimated. All the timestamps correspond to the actual local time at which data was collected. This paper did not consider the accuracy of the time stamping done by the individual sensors. However, given that the pedestrians are moving at low speeds, a coarse time synchronisation method, such as the one used in this paper, suffices. In case high-speed moving platforms are involved, a better time synchronisation method should be adopted.

### 3.2. Description of Field Test Site

**Figure 3:**
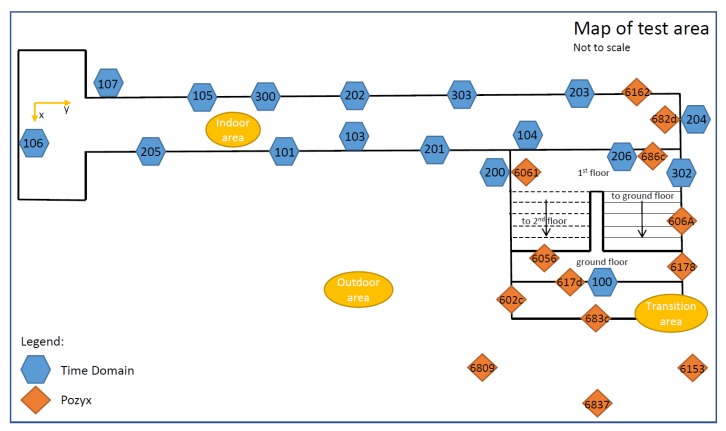
Sketch of the test site showing the locations of all the anchors. The numbers such as 106, 6809, and 682d represent the unique UWB identifiers.

The tests were carried out in a four-storey office building at Ohio State University. Figure 3 illustrates the distribution of the 17 Time Domain UWB anchors and 13 Pozyx UWB anchors. The hallway on the first floor of the building was covered with 13 Time Domain nodes and two Pozyx infrastructure nodes. The staircase leading from the first floor to the ground floor and outdoor area was equipped with four Time Domain nodes and seven Pozyx nodes. Four Pozyx devices were set up in the outdoor area in front of the building.

**Figure 4:**
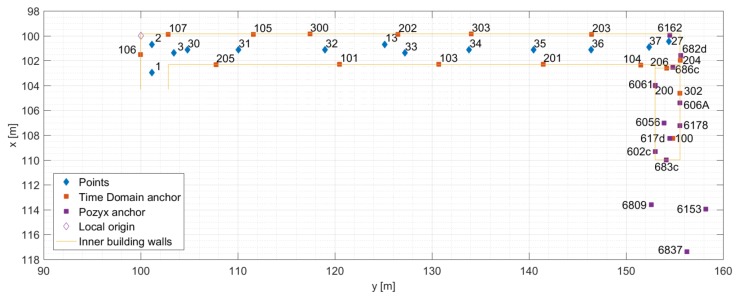
Scaled plot of the test environment with anchor UWBs and ground truth checkpoints. The numbers, such as 106, 6809, and 682d, represent the unique UWB identifiers.

Due to unfavourable observation geometry in the test area featuring a long and a narrow corridor (2.5 m wide) in which most UWB anchors lie in two parallel lines (opposite walls), special care was taken during the installation of the anchor nodes to achieve optimal geometry and visibility with regard to the dynamic nodes. Anchors were distributed in a zigzag pattern (as seen in Figure 4) with varying heights in order to reduce the occurrence of NLOS signals. A local coordinate system was set up in the building with the local origin coordinates (100,100,0) (to avoid negative 2D coordinates). The origin of the local coordinate system and the direction of the axis are shown in Figure 4. The local coordinates of all anchors were determined prior to data collection using the standard surveying techniques with 2–3 cm precision. With this UWB anchor placement, the whole test site could be covered.

The use of this test site has allowed data collection in realistic scenarios where the data quality and availability are not simulated. Data quality is affected by the environment and sensors themselves. Furthermore, during the data collection, access to the hallway and staircase was not restricted to staff and students, which may have affected the data as well. Thus, the use of this field test site offers a realistic assessment of MUPS performance capabilities in indoor areas where data quality and availability cannot be predicted due to the complexity of the environment.

### 3.3. Data Collection Procedure

In addition to determining the positions of all anchor nodes (described in Section 3.2), a set of ground truth checkpoints were set up in the hallway (blue diamonds in Figure 4). The local coordinates of checkpoints were determined with 1–2 cm precision using the standard surveying techniques. These checkpoints were used for MUPS performance assessment. Thus, the positioning errors were estimated at these points, rather than along the entire trajectory. In addition to the ground truth checkpoints, some anchor nodes were used for determining the 2D positioning error of users when they are transitioning between different areas (i.e., anchors 100, 206, 617d, 683c, and 686c set up above the doors, as shown in Figure 3).

**Figure 5:**
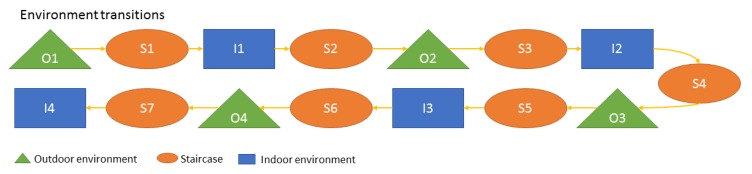
Environment transitions. *O* indicates the outdoor area, *S* indicates the staircase area, and *I* indicates the indoor area.

**Figure 6:**
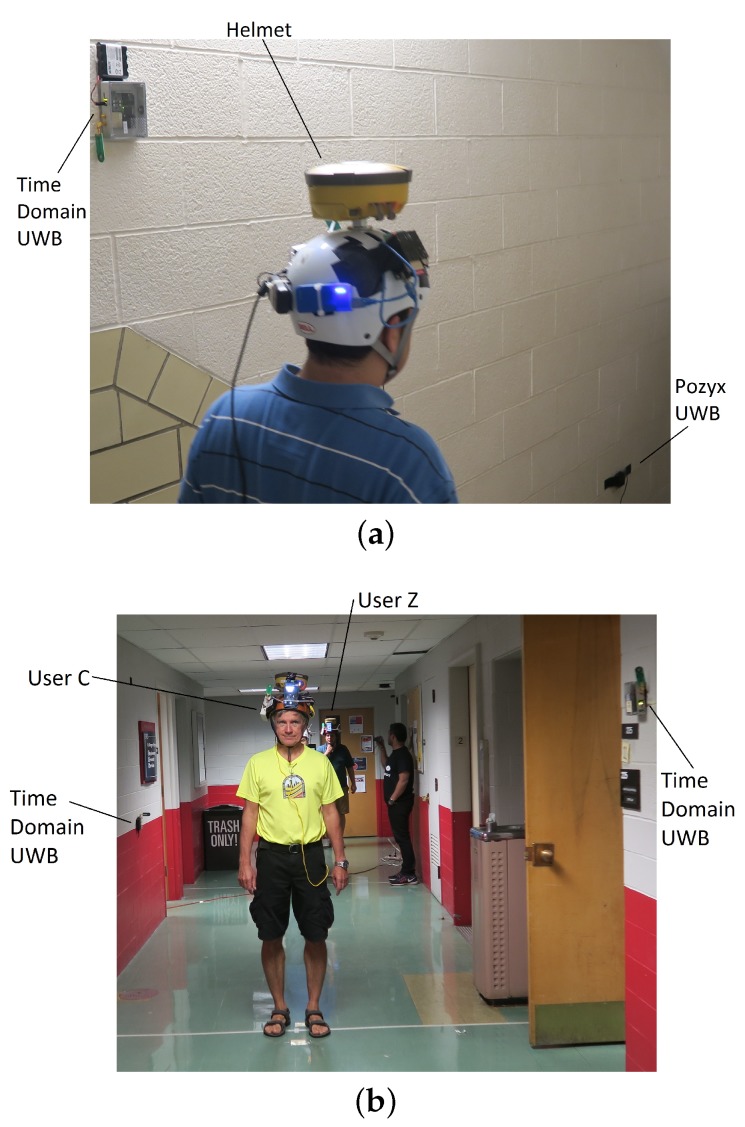
Data collection: (**a**) Staircase setup. (**b**) Hallway setup.

Dynamic nodes transitioned between three different environments: outdoors in front of the building *O*, staircase *S*, and indoors in the hallway *I*. Figure 5 shows the sequence of all environment transitions from the start to the end of the data collection (e.g., O2-S3-I2-S4 should be read: the user leaves outdoors for the second time and enters the staircase for the third time followed by the second entrance to the hallway area after which the user returns to the staircase for the fourth time). While walking along the hallway and staircase, users were stopping at checkpoints briefly (e.g., Figure 6a) to provide data for accuracy estimation.

During the experiment, data were collected for four pedestrians, each wearing prototype MUPS helmets. Due to the communication issues, two out of four pedestrians were not successful in communicating with the other two users. Hence, the analysis is presented for two of the remaining users, i.e., User Z and User C. User Z was equipped with the Time Domain device which communicated with Time Domain anchor nodes (i.e., P2I CP) and the time domain device on the User C helmet, and thus enabling P2P CP. In addition to the Time Domain device for P2P CP, User C was equipped with the Pozyx device which communicated with Pozyx anchor nodes. Figure 6 shows examples of users walking on the staircase and the hallway, including the setup of some of the infrastructure anchors.

In this paper, the goal was to determine the indoor positioning capabilities of the proposed CP MUPS. Thus, the performance along the trajectory from S1 to S2 was assessed. In future, when current CP MUPS is expanded from indoor positioning to outdoor and indoor/outdoor transition positioning, the bigger portion of the trajectory will be considered when testing the performance.

### 3.4. Data Availability in Different Environments

Data availability is an important factor in the quality of the performance. Here, the availability of Time Domain infrastructure nodes for User Z, and Pozyx infrastructure nodes for User C is analysed.

**Figure 7:**
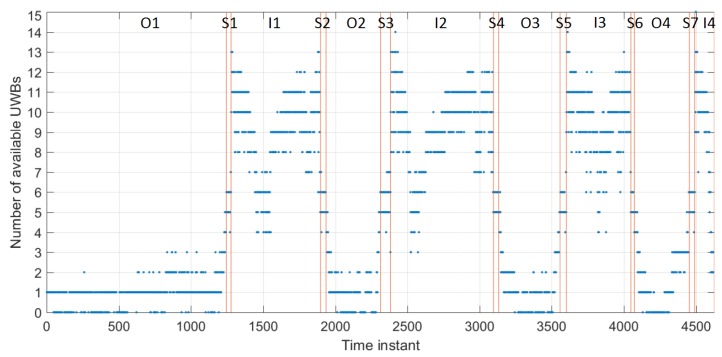
Number of available measurements from User Z to Time Domain UWB anchors. O1–O4, I1–I4, and S1–S7 denote time instants when the user was outdoors, indoors (hallway), and on the staircase, respectively.

Figure 7 shows the number of available Time Domain UWB anchors recorded by User Z in the three different environments (i.e., outdoor *O*, staircase *S*, indoor *I*). The availability outdoors is low for User Z due to not having any Time Domain devices set up outdoors (as shown in Figure 3). However, data of at least one device is available at all times (from UWB 100, which is set up at the entrance to the building). It is to be noted that the low availability of observations is due to the challenging environments. In this case, User Z could utilise available GNSS data and the CP data. User Z’s availability gets higher as the user gets closer to the staircase. Thus, the transition between the outdoor and indoor area would be possible. The availability of measurements on the staircase and hallway is good (always four or more). However, as mentioned before, due to bad geometry of such a narrow hallway, a smaller number of measurements may mean bad geometry of infrastructure nodes, which will affect the performance.

**Figure 8:**
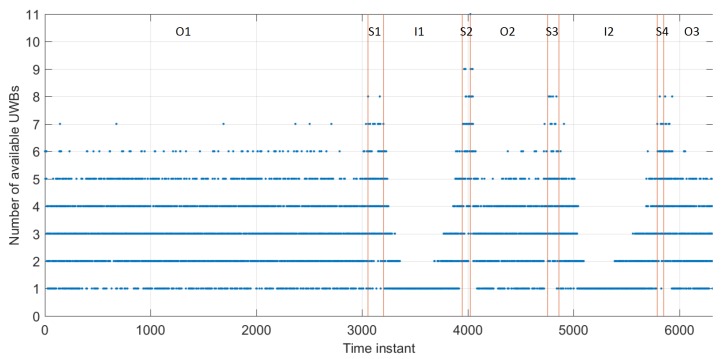
Number of available measurements from User C to Pozyx UWB anchors. O1–O3, I1–I2, and S1–S4 denote time instants when the user was outdoors, indoors (hallway), and on the staircase, respectively.

Figure 8 shows the number of available Pozyx UWB anchors recorded by User C in the three different environments. Availability of Pozyx data is good in the outdoor area due to anchors that are set up there and in the indoor/outdoor transition area (Figure 3). However, data availability becomes worse as the User C gets further away from the staircase area and into the hallway where only two anchors are set up. This will affect the User C’s positioning accuracy in the hallway area.

## 4. Positioning Framework

This section presents the mathematical framework used for developing the MUPS. Similarly to [16], a centralised, constant acceleration, cooperative Extended Kalman Filter (EKF) was implemented. Although this implementation can be generalised to more mobile nodes (as in [16]), the positioning framework for a network of two mobile nodes and $N_{IN}$ infrastructure nodes is presented here. The state vectors for two users (i.e., dynamic nodes) are simultaneously estimated. The joint state vector $X_{k}$ is given as,

(3) $$X_{k}={\left[\begin{array}{cc} \underset{1\times 9}{{\left(X_{k}^{1}\right)}^{T}} & \underset{1\times 9}{{\left(X_{k}^{2}\right)}^{T}} \end{array}\right]}^{T}$$

where $X_{k}^{1}$ and $X_{k}^{2}$ denote the User’s 1 and 2 state vectors, estimated at the time instant *k*. 3D position $r_{k}$, 3D velocity $v_{k}$, and 3D acceleration $a_{k}$ were being estimated at each time instant *k* for each dynamic node *i* where i∈1,2.

(4) $$X_{k}^{i}=\left[\begin{array}{ccc} \underset{1\times 3}{r_{k}} & \underset{1\times 3}{v_{k}} & \underset{1\times 3}{a_{k}} \end{array}\right]$$

The infrastructure nodes are static, and their positions were determined in the local coordinate system (as shown in Figure 4). The relative ranges between users enabled P2P CP, and relative ranges from users to infrastructure node enabled P2I communication. The measurement vector is denoted by $Z_{k}$ and consists of $N_{U_{1}-IN}$ relative ranges *d* between User 1 and infrastructure, $N_{U_{2}-IN}$ relative ranges *d* between User 2 and the infrastructure, and $N_{U-U}$ ranges *d* between two users.

(5) $$\underset{(N_{U-IN}+N_{U-U})\times 1}{Z_{k}}={\left[\begin{array}{ccc} \underset{1\times N_{U_{1}-IN}}{d_{k}^{U_{1}-IN}} & \underset{1\times N_{U_{2}-IN}}{d_{k}^{U_{2}-IN}} & \underset{1\times N_{U-U}}{d_{k}^{U_{1}-U_{2}}} \end{array}\right]}^{T}$$

The system model (i.e., the evolution of users’ state) is given as,

(6) $$\begin{array}{cc} X_{k} & =f(X_{k-1},\omega_{k})\\ \omega_{k} & ∼N(0,Q_{k}) \end{array}$$

where f(·) stands for the state evolution function. $X_{k-1}$ is the estimated joint state at the prior time-instant, k−1. The process noise $\omega_{k}$ is normally distributed with zero mean and covariance matrix $Q_{k}$ which consists of acceleration noise. The chosen system model is the constant acceleration model due to the use of dynamic nodes (pedestrians in this case) with low and stable accelerations. Given that, the motion model governing the state evolution function f(·) is given as,

(7) $$\begin{array}{cc} \dot{r} & =v\\ \dot{v} & =a \end{array}$$

The first equation in (7) denotes the change of position per unit of time—velocity, and the second equation denotes the change of velocity per unit of time—acceleration. As in [16], joint state evolution can be expressed as,

(8) $$X_{k}=F_{k}X_{k-1}+\omega_{k}$$

where the joint transition matrix $F_{k}$ is given in (9) and a state transition matrix $F_{k}^{i}$ for a single User *i* where i∈1,2, is given in (10). The general case for $F_{k}$ can be found in [16].

(9) $$F_{k}=\left[\begin{array}{cc} F_{k}^{1} & 0\\ 0 & F_{k}^{2} \end{array}\right]$$

(10) $$F_{k}^{i}=\left[\begin{array}{ccc} \underset{3\times 3}{I} & \underset{3\times 3}{I}\cdot \delta t & \frac{1}{2}\underset{3\times 3}{I}\cdot \delta t^{2}\\ \underset{3\times 3}{0} & \underset{3\times 3}{I} & \underset{3\times 3}{I}\cdot \delta t\\ \underset{3\times 3}{0} & \underset{3\times 3}{0} & \underset{3\times 3}{I} \end{array}\right]$$

δt denotes the time increment between two state estimates. The identity matrix is denoted by *I*. As seen in [25], the measurement model can be expressed as,

(11) $$Z_{k}=h\left(X_{k}\right)+u_{k}$$

where h(·) stands for the non-linear measurement model and $u_{k}$ stands for measurement noise. In this case, measurement noise was normally distributed with zero mean and variance matrix $R_{k}$. Non-linear measurement equations for user to infrastructure (i.e., P2I) and user to user (i.e., P2P) relative ranges are given below, respectively,

(12) $$d_{k}^{U_{i}-IN_{j}}=\sqrt{{\left(x^{j}-x_{k}^{i}\right)}^{2}+{\left(y^{j}-y_{k}^{i}\right)}^{2}+{\left(z^{j}-z_{k}^{i}\right)}^{2}}+u_{k}$$

(13) $$d_{k}^{U_{1}-U_{2}}=\sqrt{{\left(x_{k}^{1}-x_{k}^{2}\right)}^{2}+{\left(y_{k}^{1}-y_{k}^{2}\right)}^{2}+{\left(z_{k}^{1}-z_{k}^{2}\right)}^{2}}+u_{k}$$

where i∈1,2 indicates the user (i.e., mobile node) in question and $j\in 1,2,\cdots ,N_{IN}$ indicates which infrastructure node the measured range refers to. The coordinates of the infrastructure node *j* are $\left(x^{j},y^{j},z^{j}\right)$. The predicted coordinates of User 1 or 2 at time instant *k* are $\left(x_{k}^{i},y_{k}^{i},z_{k}^{i}\right)$, where i∈1,2.

The linearization of the measurement model shown in (12) and (13) with respect to any user’s estimated positions, results in,

(14) $$H_{k}^{i}=\left[\begin{array}{ccc} \underset{1\times 3}{\frac{\partial d_{k}^{i}}{\partial r_{k}^{i}}} & \underset{1\times 3}{0} & \underset{1\times 3}{0} \end{array}\right]$$

$H_{k}^{i}$ denotes a Jacobian matrix calculated as a first-order partial derivative of (12) or (13) with respect to the User’s *i* predicted state $X_{k}^{i}$. The simplified joint measurement matrix $H_{k}$ is then given as,

(15) $$\underset{\left(N_{U-IN}+N_{U-U}\right)\times \left(2\cdot 9\right)}{H_{k}}=\left[\begin{array}{cc} H_{k}^{U_{1}-IN_{1}} & H_{k}^{U_{2}-IN_{1}}\\ H_{k}^{U_{1}-IN_{2}} & H_{k}^{U_{2}-IN_{2}}\\ \vdots & \vdots \\ H_{k}^{U_{1}-IN_{N_{IN}}} & H_{k}^{U_{2}-IN_{N_{IN}}}\\ H_{k}^{U_{1}-U_{2}} & H_{k}^{U_{2}-U_{1}} \end{array}\right]$$

The general form of this matrix is given in [16]. The dimensions of the final joint measurement matrix are given in (15). The number of rows is equal to the number of available relative ranges at time instant *k*, and the number of columns in the matrix corresponds to the total number of states estimated in the joint state vector $X_{k}$. The joint measurement matrix consists of Jacobian matrices calculated for relative ranges to infrastructure nodes $1,2,\cdots ,N_{IN}$ and a Jacobian matrix for the relative range between Users 1 and 2. It is important to note that some matrices in (15) can be equal to zero. This happens whenever a measurement from a certain infrastructure node is unavailable (i.e., partial derivatives are equal to zero). In the case of P2I positioning only, the Jacobian matrices $H_{k}^{U_{1}-U_{2}}$ and $H_{k}^{U_{2}-U_{1}}$ are equal to zero. Besides the equations specified in this section, the standard EKF equations (as in [51]) can be used to estimate the position of both users. The following section shows and discusses the results achieved using this approach.

## 5. Results and Discussion

The goal of this paper was to evaluate the performance of the developed CP system and to draw a conclusion on the benefits of CP capabilities in an indoor environment where the network geometry and data availability can negatively affect performance.

To test this, experiments were performed using the positioning framework (see Section 4) on three different data sets. Firstly, User Z’s relative ranges to Time Domain infrastructure nodes (i.e., P2I) measured with a Time Domain device set up on a helmet were used. Furthermore, User C’s P2I relative ranges measured with Pozyx UWBs on the helmet to Pozyx infrastructure nodes were used. Finally, a data set made of P2I ranges for both users, with the addition of relative ranges between users (i.e., P2P+P2I) measured with Time Domain devices set up on both users’ helmets, was used. It should be noted here that the User Z was equipped with one UWB device (Time Domain) which measured both P2I and P2P ranges to User C. User C was equipped with two UWB devices, where the Time Domain device measured P2P ranges to User Z and the Pozyx device measured P2I ranges. As shown in Figure 1, there is a separation of ∼15cm between such two UWB devices, which would affect the CP estimate. However, when estimating the positions, this was not accounted for in this paper, due to the results showing average position error of magnitude ∼15–30 cm under good data availability and geometry conditions. Thus, the effect of different positions of devices was assumed to be small. Data sets used in these experiments were subsets of the collected data described in Section 3.3. Only part of the full trajectory (as seen in Figure 5) was used due to the focus of this paper on indoor navigation. The trajectory where users walked through the staircase for the first time to the hallway and back to the staircase (i.e., S1-I1-S2) was chosen. Furthermore, although the framework presented in Section 4 could be used for 3D positioning, only 2D positions were analysed in this paper, as planimetric accuracy is often more important in flat 2D environments. Additionally, the constraints of flat 2D environments can be imposed on the EKF to improve the accuracy. However, the paper did not impose any such constraints. In future work, when more data are collected on multiple building floors, the performance of the height component will be analysed. A zero-mean Gaussian error distribution with the standard deviation 1 m was assumed for all relative ranges used in the experiments. The acceleration noise was zero-mean Gaussian with the standard deviation of 1 ms^−2^. The parameters of EKF (such as *Q* and *R*) should be determined using a Kalman Filter tuning procedure. However, tuning a centralised EKF can be quite challenging. Therefore, these parameters were determined using a trial and error method. As detailed in Section 3.3, ground truth checkpoints were used to test MUPS performance in the indoor environment.

Firstly, the performance of User Z was analysed in Section 5.1 by comparing P2I and P2P+P2I CP solutions with ground truth and between each other. In Section 5.2, User C’s performance was presented and analysed similarly.

### 5.1. User Z: P2I vs. P2P+P2I

**Figure 9:**
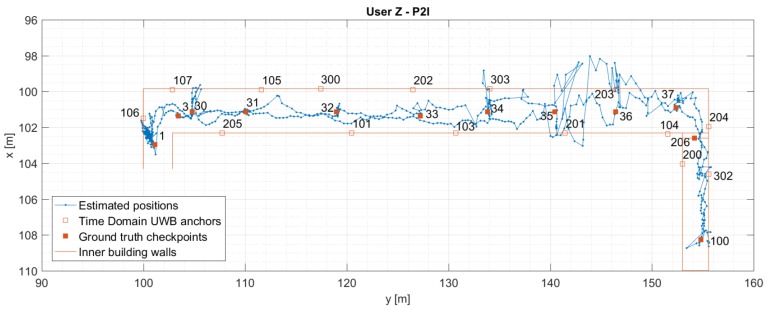
Estimated positions of the User Z for trajectory S1–I1–S2. Positions are estimated using Time Domain UWB data only (P2I EKF).

**Figure 10:**
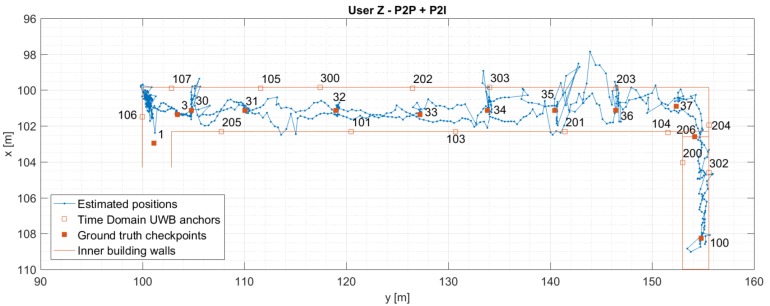
Estimated positions of the User Z for trajectory S1–I1–S2. Positions are estimated using Time Domain UWB and User C’s data (P2P+P2I EKF).

Figure 9 and Figure 10 show the estimated positions for User Z for P2I and P2P+P2I CP. Figure 9 shows an increased position error in the right part of the hallway, which was reduced once the P2P data became available (shown in Figure 10). Since the hallway is about 2.5 m wide, the positioning error is within that value most of the time; except when the position estimate wanders off beyond the walls of the hallway in the right part of the hallway. The accuracy of the position estimate on the staircase seems to be better than in the hallway, as the position estimate is always on the right side of the staircase. Both the better positioning accuracy on the staircase and left side of the hallway may be due to better geometry of nodes. Horizontal Dilution of Precision (HDOP) was used to assess the quality of the geometry. However, due to ground truth data only being available at checkpoints, HDOP (calculated based on ground truth), was also only available then.

**Figure 11:**
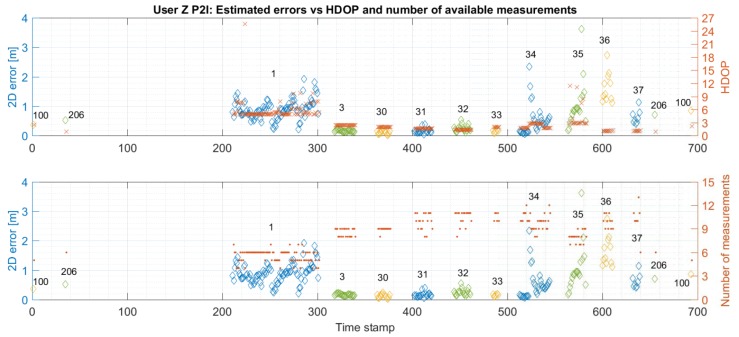
Comparison of estimated errors at each checkpoint with respective values of HDOP (upper graph) and the number of available measurements (bottom graph) for trajectory S1–I1–S2 for User Z. Positions were estimated using Time Domain UWB data only (P2I EKF).

**Figure 12:**
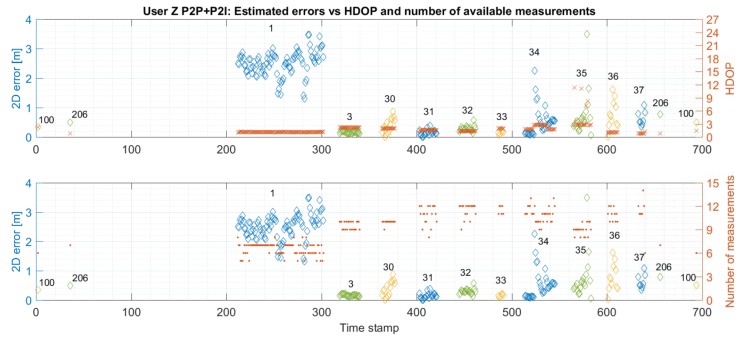
Comparison of estimated errors at each checkpoint with respective values of HDOP (upper graph) and the number of available measurements (bottom graph) for trajectory S1–I1–S2 for User Z. Positions were estimated using Time Domain UWB and User C’s data (P2P+P2I EKF).

Figure 11 and Figure 12 compare the 2D error with both the HDOP and the number of available measurements for User Z’s P2I and P2P+P2I CP solution, respectively. It should be noted that the comparison of the 2D error with the HDOP is always shown on the upper graph, while the comparison of the 2D error with the number of measurements is always shown in the bottom graph. Furthermore, the estimate of the positioning error is available only when the user was static at the checkpoints (checkpoints are marked with their IDs). Figure 11 shows that the geometry and availability of measurements are generally good (the lower the HDOP value, the better the geometry). However, an increase of error at checkpoints 1, 34, and 35 coincides with a reduced number of measurements and increased HDOP. The positioning accuracy at checkpoints 36 and 37 is similar to the positioning accuracy at checkpoints 34 and 35, despite having better geometry. As visible in Figure 11, both geometry and measurement availability improves once the User Z gets to points 36 and 37, however, the accuracy does not improve. This is probably caused by the quality of the measurements User Z receives from anchors on the staircase (anchors 100, 200, and 302) which were not available for checkpoints 34 and 35. The quality of those measurements may be affected due to the signal from nodes 200 and 302 passing twice through the walls for checkpoint 36, and once from nodes 100 and 302, and twice from node 200 for checkpoint 37. Checkpoint 1 has the largest HDOP (i.e., the worst geometry) which may be due to all UWB signals arriving from the same general direction.

Figure 12 shows that the addition of the P2P data did not significantly improve the node geometry, except for checkpoint 1. Although the geometry improved, the error increased there. Most of the time, User Z was receiving the signal from six static nodes where only two of those had direct LOS and all others had to pass through walls two or three times. Thus, the accuracy of positioning was probably affected by the reduced measurement quality.

**Figure 13:**
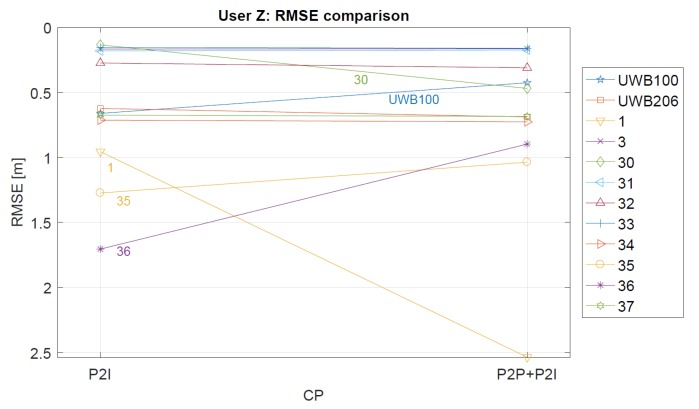
Comparison of RMSE values for P2I and P2P+P2I, for User Z. If improvement is observed, the line is oriented upwards (i.e., value of RMSE is reduced).

The statistics for User Z’s CP positioning performance is given in Table 2. For both data sets (P2I and P2P+P2I), the performance was quantified for every checkpoint. Checkpoints 3–33 show good performance with Root Mean Square Error (RMSE) values ranging from 0.13 m to 0.27 m. When geometry is bad, as indicated by a high HDOP (shown in Figure 11), RMSE values range from 0.71 m to 1.27 m for checkpoints 1, 34, and 35. Although the geometry was good at checkpoints 36 and 37, accuracy was not comparable with the accuracy at points 3–33. As discussed before, this may be a result of the reception of signals from anchor nodes on the staircase, due to which said signals pass through obstacles two or three times. Table 2 shows that the RMSE at the checkpoints 35 and 36 has been reduced for 18% and 47%, respectively, with the addition of the P2P measurements. This does not look to be true for the rest of the checkpoints, where the majority shows to have similar accuracy. However, the RMSE at checkpoint 1 was significantly increased with the addition of P2P ranges, which was not explored further in this paper. The improvement/deterioration of the positioning accuracy shown in Table 2 is illustrated in Figure 13.

User Z’s performance shows that the addition of P2P cooperative data to the P2I data can improve positioning accuracy. However, if the performance-based on P2I data only was already good (decimetre level), accuracy remained similar. In some cases, the performance was worse, which may have been due to the introduction of User C’s position uncertainty (bad geometry and low measurement availability). Section 5.2 provides more detail on this. Thus, a CP system capable of optimising the data used for position estimation at times when P2I data is good enough or P2P is necessary, would probably offer the best solution.

### 5.2. User C: P2I vs. P2P+P2I

**Figure 14:**
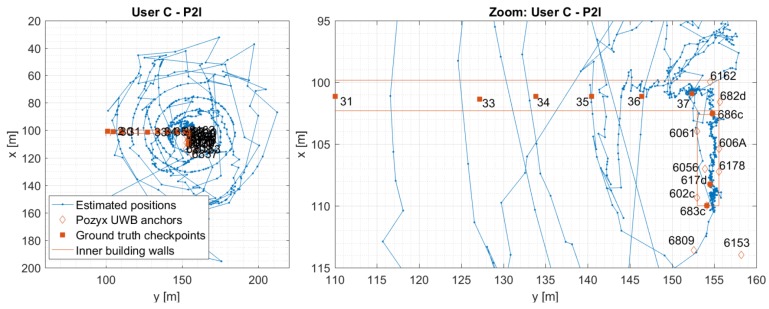
Estimated positions of the User C for trajectory S1–I1–S2. Positions are estimated using Pozyx UWB data only (P2I EKF). The graph on the right shows the zoomed view of the estimated positions.

**Figure 15:**
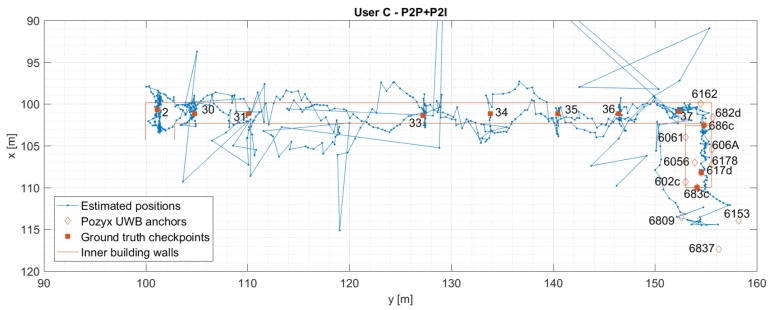
Estimated positions of the User C for trajectory S1–I1–S2. Positions are estimated using Pozyx UWB data and User Z’s data (P2P+P2I EKF).

**Figure 16:**
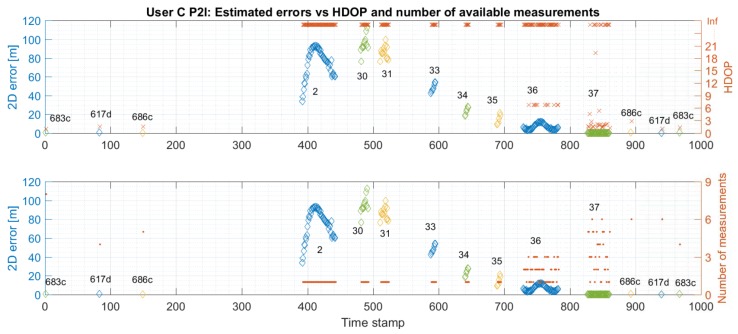
Comparison of estimated errors at each checkpoint with respective values of HDOP (upper graph) and the number of available measurements (bottom graph) for trajectory S1–I1–S2 for User C. Positions are estimated using Pozyx UWB data only (P2I EKF).

Figure 14 and Figure 15 show the estimated positions for User C for P2I and P2P+P2I CP. The position estimates of User C-based on P2I data show large errors (see Figure 14) where position estimates do not only reach outside the limits of the hallway into neighbouring rooms like for User Z. Figure 14 also shows a zoomed-in view of the estimated positions when only P2I data is used. Position estimates seem to be good on the staircase and at the beginning of the hallway (i.e., the right side of the hallway) where the number of measurements is over three. However, once the user moves on to the hallway where only one measurement is available, the position error significantly increases. This happens due to availability of data from only one infrastructure node Pozyx 682d (see Figure 16). Thus, the user seems to be walking in a spiral trajectory centred within the UWB anchor Pozyx 682d where the radius of the spiral increases/decreases as the relative range from that infrastructure node increases/decreases. Once the P2P data from User Z is added, positioning errors are reduced (shown in Figure 15).

It should be noted that the results based on P2I data, in the case of one available range measurement, are not useful since the user could be anywhere in the building. Thus, it is not appropriate to use the proposed framework for positioning based on P2I data only. Still, these results offer an insight into possible issues that can arise due to measurement unavailability and the magnitude of that effect if the system is based on one type of data (relative ranges from anchor nodes in this case). Furthermore, a significant improvement in accuracy was shown once the P2P CP data from only one additional user were fused with P2I data. To make the system more robust, a fusion of P2P+P2I data with IMU data is recommended in the case of unavailability of all relative ranging measurements.

**Figure 17:**
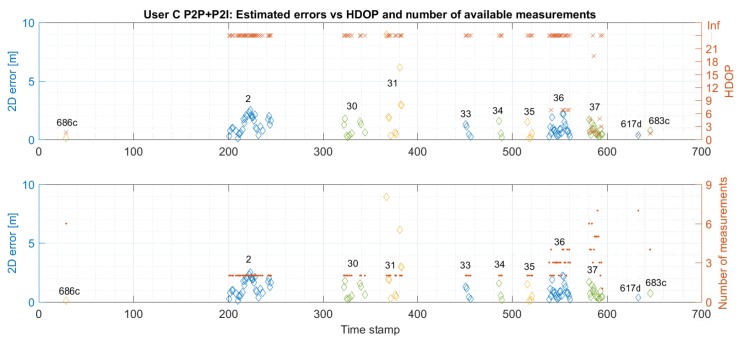
Comparison of estimated errors at each checkpoint with respective values of HDOP (upper graph) and the number of available measurements (bottom graph) for trajectory S1–I1–S2 for User C. Positions are estimated using Pozyx UWB and User Z’s data (P2P+P2I EKF).

Similarly to Section 5.1, Figure 16 and Figure 17 compare the 2D error with the calculated HDOP and the number of available measurements for User C’s P2I and P2P+P2I CP solution, respectively. Again, positioning error estimation is available only when the user was static at the checkpoints. As shown in Figure 16, the geometry seems to be good when the User C is on the staircase during the availability of multiple P2I measurements (user is on the staircase before and after he passes checkpoint 686c). The measurement availability and good geometry reflect well on positioning accuracy. The accuracy on the staircase and beginning of the hallway seems to be consistent with the one for User Z under the same conditions. However, the positioning error grows to over 100 m when only one to three P2I measurements are available, due to which the calculated HDOP approaches infinity. Once P2P measurements are added, the geometry improves on the staircase, but not in the hallway where only two to three measurements are still available (see Figure 17). This metric would improve with the addition of multiple users. Even though the geometry did not improve, communication with User Z offered the needed constraint to the one available P2I measurement in the hallway, which reduced the positioning error from ∼100 m level to mostly meter level.

**Figure 18:**
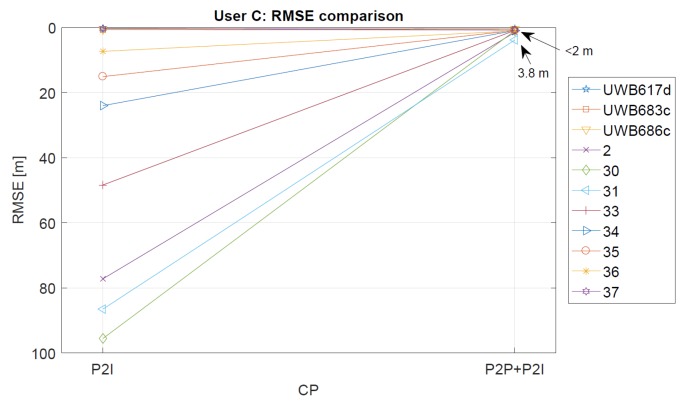
Comparison of RMSE values for P2I and P2P+P2I, for User C. If improvement is observed, the line is oriented upwards (i.e., value of RMSE is reduced).

The statistics for User C’s CP positioning performance is given in Table 3. The P2I RMSE ranges from 0.28 m to 0.64 m for checkpoints with good geometry and range availability (staircase and point 37). The points with low measurement availability and thus, very bad geometry show RMSE ranging from 7.33 m to 95.54 m (points 2–36). Once the P2P data is added, the points with good data availability and geometry have RMSE values ranging from 0.36 m to 0.80 m, and others from 0.90 m to 3.82 m. The improvement of accuracy shown in Table 3 is illustrated in Figure 18.

In the case of User C, the addition of User Z’s data made a significant impact on the resulting positioning errors for checkpoints 2–36, which were reduced on average by 95%. As for User Z, the RMSE for points that had good measurement availability and geometry was less affected by the addition of P2P ranges. However, some points did show an increase in positioning error. As in the case of User Z, this probably happens due to the addition of position uncertainty of the other user to good P2I data.

These results demonstrate some of the issues for indoor positioning systems. As explained in the earlier sections, this data was collected in a hallway at Ohio State University. The students and staff were not restricted from accessing this area, and that may have affected the quality of the measured ranges in addition to normal UWB radio measurement unavailability, possible multipath from the walls and glass surfaces, and bad geometry between the users and the UWB anchors, resulting in high HDOP values. Thus, these results offer a realistic view into the achievable performance with the presented positioning framework and quantify the effect on the performance some of the possible issues can have in indoor environments (e.g., availability of only one measurement). The benefit of CP in indoor areas where bad geometry and measurement unavailability often happens is demonstrated. Positioning errors may be further reduced with the addition of more users. However, it is also seen that when the infrastructure measurement data is good, P2P data can affect the positioning accuracy negatively. This is one of the drawbacks of CP but could be avoided with network optimisation and possibly with the addition of multiple users. It may be possible to further improve the localisation accuracy by imposing additional constraints, such as information derived from indoor maps. However, this paper assumes that such information is not available to the user, and hence not used in this paper.

## 6. Conclusions

The data used in this paper were collected as a part of a joint IAG and FIG working groups on a “multi-sensor systems” benchmarking measurement campaign. Although this campaign aimed to explore CP of different platforms (i.e., vehicles, bicyclists, and pedestrians) in GNSS-denied/challenged environments, in this paper, we mainly focused on pedestrian CP navigation in a GNSS-denied environment (i.e., indoors). A specially designed prototype of MUPS was used for data collection. Every prototype was equipped with a variety of sensors. In this paper, we focused on UWB-based CP between two pedestrians navigating jointly within a neighbourhood. An overview of the field experimental schemes, set-ups, characteristics, and sensor specifications, along with the main results for the positioning with two different UWB systems were presented. We demonstrated and discussed the benefits of the MUPS. The test set-ups for the UWB-based CP system in the different test scenarios have proved that they are suitable and practicable. The results show that such a system is able to achieve decimetre-level accuracy for Peer-to-Infrastructure (P2I) CP in conditions of good network geometry and measurement availability. It has to be noted that the measurement collection was carried out during regular office hours where people were moving around freely, which provided realistic performance estimates in regard to good or bad environment conditions. We have also shown that under unfavourable conditions (i.e., bad geometry, low measurement availability), accuracy degrades significantly. If the measurements are available and the network geometry is not good, the experiments show degradation of accuracy from decimetre level to meter level. However, when the measurement availability is very low (i.e., one to two measurements), the accuracy of P2I CP was degraded from decimetre level to average 50-meter level, which has been shown as the biggest disadvantage of the proposed framework due to its inability to constrain one available measurement. That constraint was offered with the additional usage of Peer-to-Peer (P2P) ranges between the two users, which resulted in a significant reduction of positioning error. Had some constraints been introduced to the positioning system (i.e., no running through walls, a pedestrian cannot move 10 m in 1 s), this improvement would not be so severe. However, in the absence of such constraints, the average improvement of 95% does indicate the benefit of CP even with just two users. In cases of good measurement availability and bad network geometry, the addition of P2P to P2I ranges (i.e., P2P+P2I CP) has improved accuracy from meter level to sub-meter levels. When only a few (one or two) measurements were available, positioning accuracy improved from average 50 m level to the average meter level. However, when geometry and measurement availability conditions were good, the addition of P2P CP sometimes negatively affected the performance. Thus, in future, MUPS needs to be capable of network optimisation based on the criteria of geometry and measurement availability. Furthermore, it is expected that even better performance is achievable if communication between multiple dynamic platforms is available. Although the presented CP framework is theoretically scalable to an arbitrary number of nodes, in practice, the network size would be limited by the communication capabilities of the UWB sensors. With the increase in the number of users, we would expect more communication outages among the nodes. However, the proposed EKF is capable of handling large networks in terms of computational requirements.

In future, the integration of other signals-of-opportunities with the existing positioning solution will be tested. In this case, the most prominent signal-of-opportunity, Wi-Fi, will be used for the indoor positioning. We will further focus on analyses of the MUPS positioning performance in the transitional environment (e.g., between outdoor and indoor), which will be enabled by integrating the presented positioning framework with GNSS observations. For that purpose, a modified and extended CP algorithm is currently being developed. Apart from absolute localisation of the users, dead reckoning with the inertial sensors is a goal of future investigations. In addition to the inertial sensors, the use of other smartphone sensors will also be investigated (e.g., the use of a barometric pressure sensor for improving 3D localisation of the users in a multi-storey building). This paper did not consider any network security-related issues in the cooperative positioning system. From a practical perspective, such issues may affect the overall performance and integrity of the system. The network security in cooperative systems will be considered in future research. 

## Figures and Tables

**Table 1:** Specifications of Time Domain ([45,46]) and Pozyx * [47] UWB systems.

Item	Time Domain	Pozyx
Dimensions	76×80 mm	71.75×58 mm
Weight	58 g	12 g
Ranging Accuracy	∼2–3 cm	∼10 cm
Other sensors	-	9-axes IMU
		Pressure sensor

* An older version of the creator series was used.

**Table 2:** User Z’s statistics for P2I and P2P+P2I CP positioning.

User Z	P2I					P2P+P2I				
Point ID	RMSE	Avg	Max	Avg	Max	RMSE	Avg	Max	Avg	Max
	[m]	[m]	[m]	HDOP	HDOP	[m]	[m]	[m]	HDOP	HDOP
UWB 100	0.66	0.61	0.86	2.31	2.47	0.43	0.42	0.50	1.87	2.27
UWB 206	0.62	0.62	0.71	0.92	0.92	0.65	0.64	0.78	0.86	0.87
1	0.96	0.90	1.92	5.83	25.60	2.54	2.50	3.48	1.12	1.20
3	0.17	0.16	0.22	2.43	2.43	0.16	0.16	0.22	2.21	2.22
30	0.13	0.12	0.24	1.99	2.00	0.47	0.40	0.87	1.97	1.98
31	0.18	0.16	0.39	1.62	1.65	0.17	0.15	0.39	1.61	1.65
32	0.27	0.25	0.55	1.36	1.41	0.31	0.30	0.57	1.36	1.40
33	0.16	0.15	0.20	1.95	1.96	0.16	0.16	0.21	1.93	1.94
34	0.71	0.51	2.34	2.24	2.86	0.73	0.53	2.25	2.24	2.85
35	1.27	1.03	3.62	4.36	11.40	1.04	0.73	3.49	4.30	11.35
36	1.71	1.63	2.74	1.12	1.22	0.90	0.77	1.61	1.09	1.17
37	0.68	0.64	1.14	1.09	1.21	0.68	0.64	1.14	0.87	1.21

**Table 3:** User C’s statistics for P2I and P2P+P2I CP positioning.

User C	P2I					P2P+P2I				
Point ID	RMSE	Avg	Max	Avg	Max	RMSE	Avg	Max	Avg	Max
	[m]	[m]	[m]	HDOP	HDOP	[m]	[m]	[m]	HDOP	HDOP
UWB 617d	0.45	0.44	0.52	1.33	1.61	0.36	0.36	0.36	1.05	1.05
UWB 683c	0.64	0.63	0.75	1.20	1.35	0.74	0.74	0.74	1.35	1.35
UWB 686c	0.61	0.52	0.84	2.23	2.89	0.41	0.34	0.57	1.57	1.57
2	77.22	75.77	93.29	Inf *	Inf	1.49	1.33	2.50	Inf	Inf
30	95.54	95.13	112.53	Inf	Inf	0.99	0.83	1.76	Inf	Inf
31	86.52	86.29	99.78	Inf	Inf	3.82	2.78	8.96	Inf	Inf
33	48.35	48.20	54.19	Inf	Inf	0.90	0.80	1.29	Inf	Inf
34	24.01	23.72	28.25	Inf	Inf	0.95	0.75	1.55	Inf	Inf
35	15.09	14.38	21.22	Inf	Inf	0.91	0.71	1.49	Inf	Inf
36	7.33	6.73	12.24	Inf	Inf	1.07	0.88	2.21	Inf	Inf
37	0.28	0.27	0.46	Inf	Inf	0.80	0.70	1.67	Inf	Inf

* HDOP approaches infinity (relatively to normal HDOP values) when only 1–3 measurements are available.
